# Swine Conjunctivitis Associated with a Novel Mycoplasma Species Closely Related to *Mycoplasma hyorhinis*

**DOI:** 10.3390/pathogens10010013

**Published:** 2020-12-25

**Authors:** Isabel Hennig-Pauka, Christoph Sudendey, Sven Kleinschmidt, Werner Ruppitsch, Igor Loncaric, Joachim Spergser

**Affiliations:** 1 Field Station for Epidemiology in Bakum, University of Veterinary Medicine Hannover, 49456 Bakum, Germany; isabel.hennig-pauka@tiho-hannover.de; 2 Tierärztliche Gemeinschaftspraxis Büren FGS-GmbH, 33142 Büren, Germany; C.Sudendey@fgs-bueren.de; 3 Lower Saxony State Office for Consumer Protection and Food Safety, Food and Veterinary Institute Braunschweig/Hannover, 30173 Hannover, Germany; Sven.Kleinschmidt@laves.niedersachsen.de; 4 Institute of Medical Microbiology and Hygiene, Austrian Agency for Health and Food Safety, 1096 Vienna, Austria; werner.ruppitsch@ages.at; 5 Institute of Microbiology, University of Veterinary Medicine Vienna, 1210 Vienna, Austria; igor.loncaric@vetmeduni.ac.at

**Keywords:** ammonia, conjunctivitis, lymphohistiocytic inflammation, *Mycoplasma* sp. nov., myo-inositol pathway, swine

## Abstract

Conjunctivitis in swine is a common finding, usually considered to be a secondary symptom of respiratory or viral systemic disease, or a result of irritation by dust or ammonia, or of local infections with *Mycoplasma* (*M.*) *hyorhinis* or chlamydia. In three unrelated swine farms in Germany with a high prevalence of conjunctivitis, a novel mycoplasma species, tentatively named *Mycoplasma* sp. 1654_15, was isolated from conjunctival swabs taken from affected pigs. Although 16S rRNA gene sequences shared highest nucleotide similarities with *M. hyorhinis*, matrix-assisted laser desorption ionization-time of flight (MALDI-TOF) mass spectrometry, partial *rpoB* sequencing, and comparative whole genome analyses indicated the identification of a novel species within genus *Mycoplasma*. Noticeable differences between *Mycoplasma* sp. 1654_15 and *M. hyorhinis* were the lack of a *vlp* locus and the presence of a myo-inositol pathway in the genome of strain 1654_15. Since myo-inositol might be used as an alternative energy source by this pathogen on the conjunctival surface, robust colonization by outcompeting other bacteria could be the consequence. In summary, abundant isolation of *Mycoplasma* sp. 1654_15 from the conjunctiva of affected pigs, its close relationship to *M. hyorhinis*, and identification of a panel of coding sequences (CDSs) potentially associated with virulence and pathogenicity suggested a local eye disease caused by a so far unknown, highly specialized mycoplasma species.

## 1. Introduction

Mycoplasmas are characterized by a lack of cell wall, and a small cell and genome size, resulting in limited metabolic capabilities and intimate parasitic lifestyle depending on nutrients from their hosts [1,2,3]. Four *Mycoplasma* (*M*.) species have been commonly implicated in causing disease in swine, including *M. hyopneumoniae*, which is the primary pathogen of porcine enzootic pneumonia (EP), *M. hyosynoviae*, which may lead to arthritis in growing pigs, *M. suis*, the cause of porcine eperythrozoonosis, and *M. hyorhinis*, a common inhabitant of the upper respiratory tract associated with polyserositis and arthritis in younger pigs [4,5]. Other porcine mycoplasmas such as *M. flocculare* and *M. hyopharyngis* are commonly described as commensals residing harmless in the porcine respiratory tract [1].

*M. hyorhinis* is transmitted shortly after birth from colonized sows to their offspring which are then a source of transmission to other piglets in the nursery period resulting in up to 98% pigs being colonized in the postweaning period [5]. It is unknown, under which circumstances *M. hyorhinis* spreads from the respiratory tract to serosal and joint tissues. However, stress and coinfections have been considered important trigger factors for disease development [6,7]. In addition, differences in virulence of *M. hyorhinis* strains have been described previously [1,8] but specific virulence factors contributing to systemic infection have not been identified so far. However, it has been suggested that variable surface-exposed lipoproteins (Vlp’s) are likely the cause of inflammatory reactions in systemically infected pigs [9]. Although the main clinical picture of systemic infection is polyserositis and arthritis, *M. hyorhinis* has occasionally been associated with other diseases such as pneumonia [6,7,8,10,11], otitis and eustachitis [12,13,14], abortion [15], and conjunctivitis [16]. In general, conjunctivitis in swine is not considered a disease of high economic importance and, in consequence, diagnostic investigations are rather limited, mostly relating swine conjunctivitis to systemic viral or local chlamydia infections, and to irritating environmental substances or foreign bodies in the eye [16,17]. Although various mycoplasma species have been implicated in causing conjunctivitis in several other animals [18,19,20,21,22], only two case reports on mycoplasma-associated swine conjunctivitis have been published so far. While *M. hyorhinis* has been identified as causative agent in one of these reports [16], unclassified mycoplasma-like organisms adhering superficially to epithelial cells or located intracellularly within membrane-bound vacuoles have been suggested as cause of chronic lymphoplasmacytic conjunctivitis in the other investigation [23]. Furthermore, examination of conjunctival samples from piglets for mycoplasmas resulted in the isolation of *M. hyorhinis*, *M. flocculare* and an unidentifiable mycoplasma species [24]. In the most recent case report conjunctivitis in swine was diagnosed in three wean-to-finish farms in the United States with clinical signs observed in two months old pigs that persisted until the pigs were 5–6 months of age [16].

Here, we report a similar field observation of swine conjunctivitis in three unrelated farms in Germany. Respiratory disease was the major concern in two producers, while in one farm conjunctivitis alone was the focus of measures to improve animal welfare. In all three farms a novel mycoplasma species, tentatively named *Mycoplasma* sp. 1654_15, was abundantly isolated from conjunctival swabs taken from affected pigs. Although 16S rRNA gene sequences of mycoplasma isolates shared highest nucleotide similarities with *M. hyorhinis* (98.90–99.11%), matrix-assisted laser desorption ionization-time of flight (MALDI-TOF) mass spectrometry, partial *rpoB* sequencing, and comparative whole genome analyses highly suggested that *Mycoplasma* sp. 1654_15 represents a new species within genus *Mycoplasma*.

## 2. Results

### 2.1. Macroscopic and Histopathological Findings

**Figure 1:**
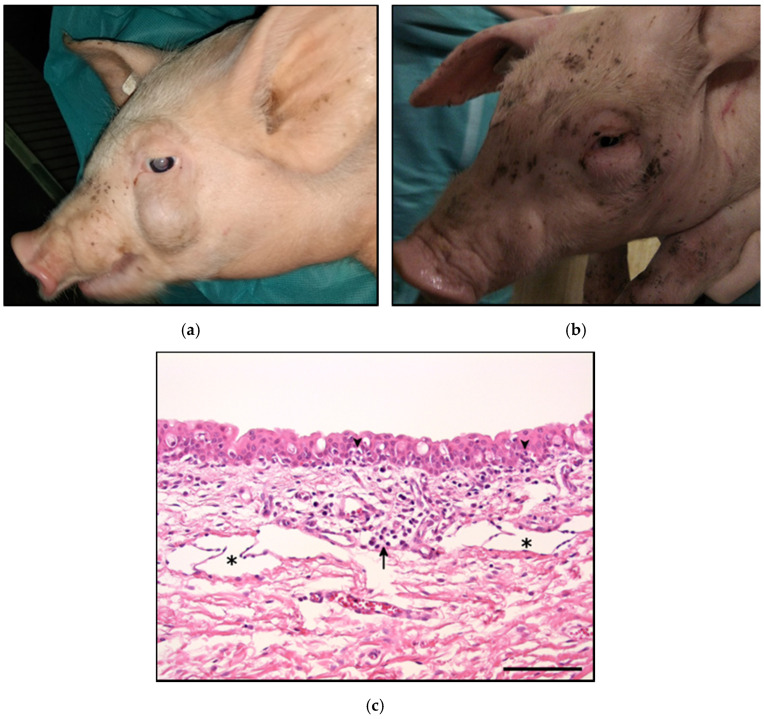
Pigs from farm A displaying various forms of swellings around the eyes (**a**) and conjunctivitis (**b**). Histological findings in the tunica conjunctiva palpebrarum (**c**). Haematoxylin and eosin (HE). Bar = 100 µm. Mild multifocal lymphohistiocytic conjunctivitis (arrow) with moderate lymphangiectasis (asterisks). Few lymphocytes migrate into squamous epithelial layer (arrow heads). Lamina propria shows a mild oedema.

In farm A several pigs showed swelling around the eyes without (Figure 1a) or with obvious signs of conjunctivitis (Figure 1b). Macroscopic swellings were assessed to be the consequence of inflammatory subepithelial oedema due to a dilatation of lymphatic vessels after a fluid-pressure-related drainage disorder. Main findings during necropsy of four pigs were a catarrhal to purulent bronchopneumonia and sporadic detection of *Streptococcus* (*S.*) *suis* and *Glaesserella* (*G.*) *parasuis* in the bronchi. One pig positive for *S. suis* in bronchi, lung tissue, and the nose was also found positive for the porcine reproductive and respiratory syndrome virus PRRSV in the lung. In all necropsied pigs *M. hyorhinis* was detected in the nose using PCR. Histological examination of eye alterations in one pig resulted in a multifocal lymphohistiocytic inflammation mainly around the margin of the eyelids (Figure 1c). Findings in eye swabs, sampled from 15 animals, are summarized in Table 1. Ammonia concentrations in farm A were found to be 30–50 ppm.

**Figure 2:**
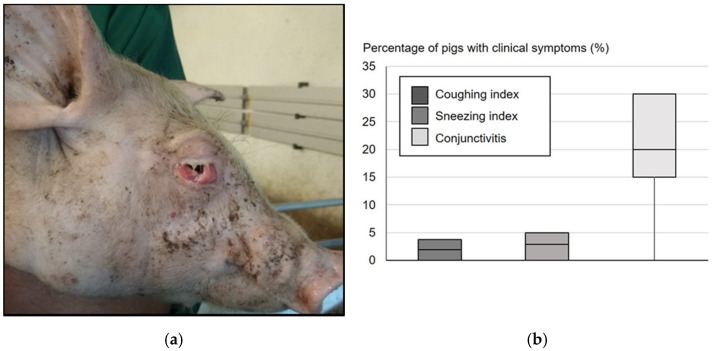
Severe conjunctivitis in a pig from farm B (**a**) and percentage of pigs with clinical symptoms at the age of 9–10 weeks in farm B (**b**). Boxplots show minimal and maximal values. The box represents the interquartile range including the median.

In farm B, several older pigs in the nursery units showed purulent conjunctivitis or an oedematous conjunctiva with prolapsus of the third eye lid (Figure 2a). The prevalence of conjunctivitis in pigs at the age of 9–10 weeks was 20% in median (Figure 2b). Main macroscopic findings in three of six pigs sent for necropsy were endocarditis, peritonitis, or fibrinous pleuritis. In one animal presenting alterations in all three body sites *G. parasuis* serotype 13, *M. hyorhinis* and PRRSV were detected in the lung. In addition, *M. hyorhinis* and *G. parasuis* were detected in the abdominal cavity by PCR. Results of bacteriological examination of eye samples are presented in Table 1. Ammonia concentrations in farm B were between 20–26 ppm.

**Figure 3:**
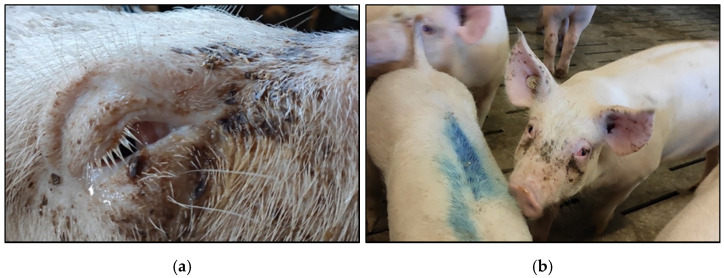

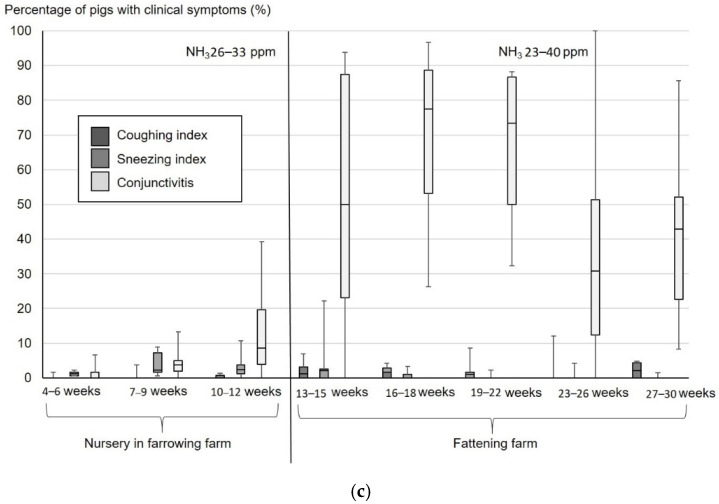
Clinical signs in pigs from farm C presenting conjunctivitis with ocular discharge (**a**) resulting in dusty tear trails (**b**). Percentage of pigs with clinical symptoms in different age groups in farm C (**c**). Boxplots show minimal and maximal values. The box represents the interquartile range including the median. 1503 piglets in the age of 4–12 weeks were examined in the nursery unit of the farrowing farm, while 1847 pigs were examined in the fattening farm. The range of ammonia concentration in air measured during the farm visit is indicated.

In farm C, the predominant symptom was conjunctivitis with serous ocular discharge (Figure 3a) resulting in dusty tear trails below the eyes in most pigs (Figure 3b). Prevalence of conjunctivitis increased with age (Figure 3c). Two pigs with body weights of 23 kg and 26 kg sent for necropsy both showed severe fibrinous pleuritis, pericarditis, and peritonitis, as well as a catarrhal to purulent bronchopneumonia. Lymph nodes examined for PCV2 by PCR were negative. Eye samples collected in the nursery unit of the farrowing farm were negative for mycoplasmas and chlamydia (data not shown). Results of bacteriological examination for eye samples taken from pigs in the fattening farm are presented in Table 1. Relatively high ammonia concentrations (23–40 ppm) were measured in all compartments (Figure 3c).

### 2.2. Characterization of Mycoplasma sp. Isolates Cultivated from Eye Swabs

In total, 35 conjunctival swabs (farm A, *n* = 15; farm B, *n* = 8; farm C, *n* = 12) were investigated for potential pathogens using conventional cultivation procedures and PCR. In 33 swab samples (94%) mycoplasmas were abundantly present, shown by colony formation on agar plates inoculated with the highest sample dilution (10^−5^), from which single colonies were selected for further analyses. In six samples (17%), however, overgrowth by bacteria prevented the establishment of pure mycoplasma cultures required for species identification. Only two samples (6%) were tested negative for mycoplasmas by cultivation. Pure cultures derived from single colonies were then subjected to species identification by MALDI-TOF mass spectrometry (MS). Despite using a large in-house mycoplasma library [25] including reference spectra or main spectrum profiles (MSP) from all known cultivable porcine mycoplasma species, mycoplasma isolates from farm A remained undiagnosed, producing best log scores <1.70 (e.g., best log score of 1.30 to *M. hyorhinis*), which was considered unacceptable for identification. Consequently, an MSP from strain 1654_15 was generated and included in the database, resulting in log score values far above 2.00 for all mycoplasma isolates from farm A, thereby providing first evidence that the mycoplasmas isolated are members of the same but undescribed porcine mycoplasma species, tentatively named *Mycoplasma* sp. 1654_15 (referring to the strain that has been genome sequenced). Similar results were obtained with mycoplasma isolates from farm B and C all producing log scores above 2.00 to strain 1654_15 (farm A) as well as to strains 2184_1 (farm B) and 338_4 (farm C), from which MSPs were consecutively generated and included in the library in order to cover the natural spectrometric diversity of a given species (Supplementary Figure S1).

**Figure 4:**
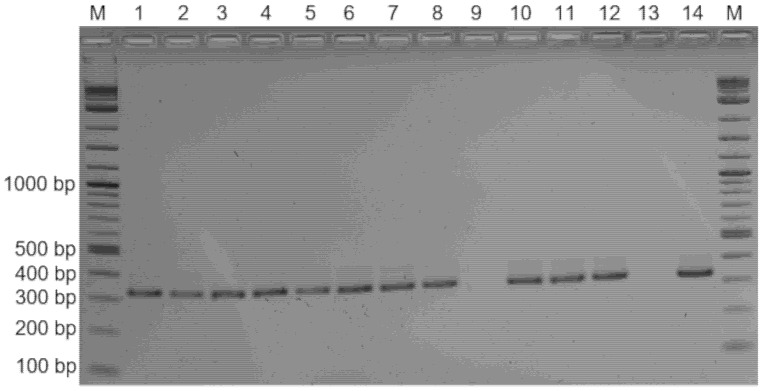
Molecular detection and identification of *Mycoplasma* sp. 1654_15 in samples including those overgrown by bacteria or from which no mycoplasmas were isolated, using PCR targeting a species–specific region of the *p37* gene. Lane 1: sample 1654_2, 2: 1654_3, 3: 1654_5, 4: 1654_10, 5: 1654_11, 6: 1654_14, 7: 2184_7, 8: 2184_8; 9: negative control (water), 10: 1654_1, 11: 2184_2, 12: 338_9, 13: *M. hyorhinis* BTS-7^T^, 14: strain 1654_15, M: molecular weight marker.

After genome sequencing of strain 1654_15 and phylogenetic positioning of nine selected mycoplasma strains isolated in this study (see below), revealing that *Mycoplasma* sp. 1654_15 represents a new mycoplasma species that is closely related to *M. hyorhinis*, a PCR for the specific detection of the new mycoplasma species was developed targeting a unique sequence in the upstream region of its *p37* gene. Stored DNA from swabs were tested by this newly established PCR resulting in the detection of *Mycoplasma* sp. 1654_15 in all samples including those from which no mycoplasmas were isolated (*n* = 2) or in which species identification of cultivated mycoplasmas was hampered by bacterial overgrowth (*n* = 6) (Table 1, Figure 4).

Samples were further tested for *M. hyorhinis* and *Chlamydia* spp. using PCR. Only in three samples (one from each farm) were *M. hyorhinis* detected by PCR targeting a *M. hyorhinis*-specific sequence of the *p37* gene. Chlamydia was present in eight samples (farm A, *n* = 3; farm B, *n* = 2, farm C, *n* = 3) which were all identified as *Chlamydia suis* by amplicon sequencing. From eight samples, bacteria commonly residing on the porcine skin (*St. hyicus*, *St. chromogenes*) as well as *Escherichia coli* were isolated, which were considered as naturally occurring contaminants in samples taken from the porcine eye (Table 1). *G. parasuis* was not detected in any of the samples examined.

Minimum inhibitory concentration (MIC) values of three selected *Mycoplasma* sp. 1654_15 strains from each farm were determined using the microbroth dilution method. Results are summarized in Table 2, demonstrating identical MIC values for all 3 strains from each case. MIC values of quality control strain *M. hyorhinis* BTS-7^T^ were in accordance with those described previously [26]. Identical or similarly low MIC values of *Mycoplasma* sp. 1654_15 strains and *M. hyorhinis* BTS-7^T^ were obtained for tiamulin, oxytetracycline, gentamicin, spectinomycin, and florfenicol. In contrast, MIC values of novel mycoplasma strains for macrolides (tylosin tartrate– 8–16 µg/mL, tilmicosin and tulathromycin->64 µL/mL), lincomycin (2–4 µg/mL) and enrofloxacin (4–8 µg/mL) were considerably high compared to those obtained for *M. hyorhinis* BTS-7^T^ (Table 2) but were in accordance with an increasing resistance of more recent *M. hyorhinis* strains to macrolides, lincosamides, and fluoroquinolones observed in Europe [26,27].

**Figure 5:**
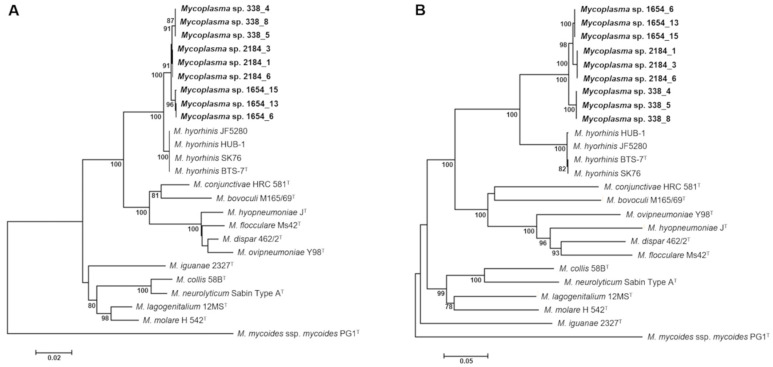
Maximum likelihood trees based on 16S rRNA gene sequences (**A**) and partial *rpoB* gene sequences (**B**) of nine selected *Mycoplasma* sp. 1654_15 strains (three from each farm) and members of the Hyopneumoniae–Neurolyticum cluster. *M. mycoides* ssp. *mycoides* was used as outgroup. Numbers at nodes represent bootstrap confidence values (1000 replications). Only values ≥75% are shown. Bar, number of substitutions per nucleotide position.

Since we were unable to identify the mycoplasma isolates by MALDI-TOF MS, two phylogenetic marker genes, the 16S rRNA and partial *rpoB* gene, were analyzed for classification and phylogenetic positioning of selected *Mycoplasma* sp. 1654_15 strains isolated in this study. Comparison of almost complete 16S rRNA gene sequences (approximately 1450 nt) demonstrated that *Mycoplasma* sp. 1654_15 is a member of the Hyopneumoniae–Neurolyticum cluster of genus *Mycoplasma*. 16S rRNA gene sequences of *Mycoplasma* sp. 1654_15 strains were only slightly different exhibiting similarity values of 99.31–99.86% to each other. In addition, they were shown to be closely related to *M. hyorhinis* expressed by highest sequence similarity values of 98.90–99.11% to *M. hyorhinis* strains, indicating a limited resolution capacity of the 16S rRNA gene precluding its utilization for accurate identification at the species level. The phylogenetic tree constructed confirmed the close relationship of *Mycoplasma* sp. 1654_15 to *M. hyorhinis*, but also illustrates a clear phylogenetic separation of both species strain cohorts supported by a bootstrap value of 100% (Figure 5A).

Partial *rpoB* gene sequences (approximately 1,780 nt) of *Mycoplasma* sp. 1654_15 strains were highly similar with calculated similarity values of 98.59% (338_4, 338_5, 338_8), 99.33% (2184_1, 2184_3, 2184_6), and 100% (1654_6, 1654_13) to the partial *rpoB* gene sequence of strain 1654_15. When partial *rpoB* sequences were compared to those of related *Mycoplasma* species (Hyopneumoniae–Neurolyticum cluster), again highest sequence similarity values of 89.16–89.44% to *M. hyorhinis* strains were observed. Since calculated similarity values were below the proposed cut-off for *rpoB* gene sequence-based identification of bacteria including mycoplasmas [28,29,30], the observed sequence differences emphasized that *Mycoplasma* sp. 1654_15 and *M. hyorhinis* represent phylogenetically closely related but distinct species in genus *Mycoplasma* (Figure 5B).

A total of 480,737 Nanopore long reads (mean length 8,240 bp) and 327,946 paired Illumina short reads (mean length 213 bp), sequenced from strain 1654_15, were hybrid assembled resulting in a complete circular chromosome with a genome size of 923,061 bp and an overall G+C content of 26.1%. Based on annotation with PGAP, the genome contained 766 predicted protein coding sequences (CDS), one copy of complete 5S, 16S, and 23S rRNA genes, 30 tRNAs, and 3 noncoding RNAs (ncRNAs). A considerable number of CDS products (*n* = 183, 24%) were annotated as hypothetical proteins, and a total of 19 IS3 family transposase CDSs were found in the genome.

Genomic relatedness between strain 1654_15 and *M. hyorhinis* SK76, HUB-1, and JF5820 were determined by calculating average nucleotide identity based on BLAST algorithm (ANIb) or MUMmer (ANIm), tetranucleotide signature correlation index (TETRA), and digital DNA-DNA hybridization (dDDH) scores. ANIb and ANIm values of strain 1654_15 to *M. hyorhinis* SK76, HUB-1, and JF5820 were 83.30/85.88, 82.83/85.54, and 83.04/85.43%, respectively, and thus far below the proposed species delineation threshold of 95-96% [31]. In contrast, paired ANIb scores between *M. hyorhinis* SK76, HUB-1, JF5820 and four further *M. hyorhinis* strains available in the JSpeciesWS’ genome database ranged between 99.06 and 99.40%, and a similar range (99.26–99.91%) were observed for ANIm scores. Furthermore, TETRA analysis yielded correlation coefficients of 0.971, 0.968, and 0.970 when strain 1654_15 was compared to *M. hyorhinis* SK76, HUB-1 and JF5820, and thus were below correlation coefficients of >0.99 calculated for genomes within the species boundary [31]. Score sets (ANIb, ANIm, TETRA) were supported by low in silico dDDH values between strain 1654_15 and *M. hyorhinis* strains (SK76:25.3%; HUB-1–25%; JF5820–25%) altogether strongly suggesting that *Mycoplasma* sp. 1654_15 represents a novel taxon in genus *Mycoplasma*.

**Figure 6:**
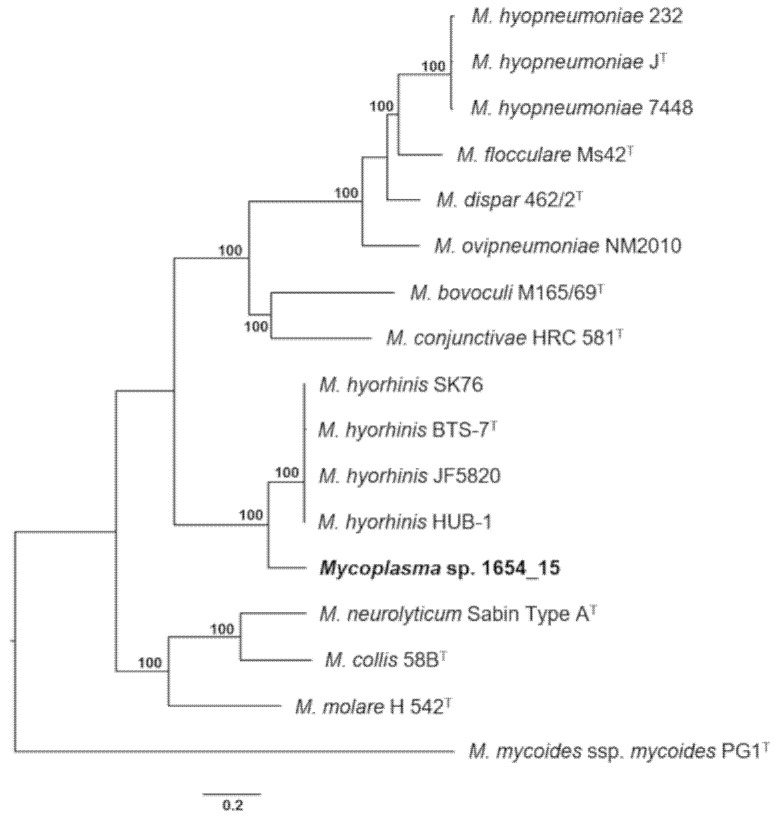
Phylogenetic tree based on whole genome comparison of strain 1654_15 and selected members of the Hyopneumoniae–Neurolyticum cluster using Randomized Axelerated Maximum Likelihood (RAxML) within the codon tree pipeline at PATRIC, analyzing 261 single-copy coding sequences. *M. mycoides* ssp. *mycoides* was used as outgroup. Numbers at nodes represent confidence values of 100 rounds of fast bootstrapping (only 100% are shown). Bar, number of substitutions per site.

A phylogenetic tree based on whole genome comparison of strain 1654_15 and selected members of the Hyopneumoniae–Neurolyticum cluster was generated using the codon tree pipeline in Pathosystems Resource Integration Center (PATRIC), which used cross-genera protein families (PGFams) as homology groups and analyzed 261 single-copy coding sequences resulting in alignment of 93,615 amino acids and 280,845 nucleotides. Overall, the phylogenetic tree constructed corroborated the results obtained from phylogenetic analyses of the 16S rRNA gene and partial *rpoB* sequences (Figure 5), confirming the close relatedness of *Mycoplasma* sp. 1654_15 to *M. hyorhinis* and their basal positioning to the Hyopneumoniae clade of the Hyopneumoniae–Neurolyticum cluster (Figure 6).

**Figure 7:**
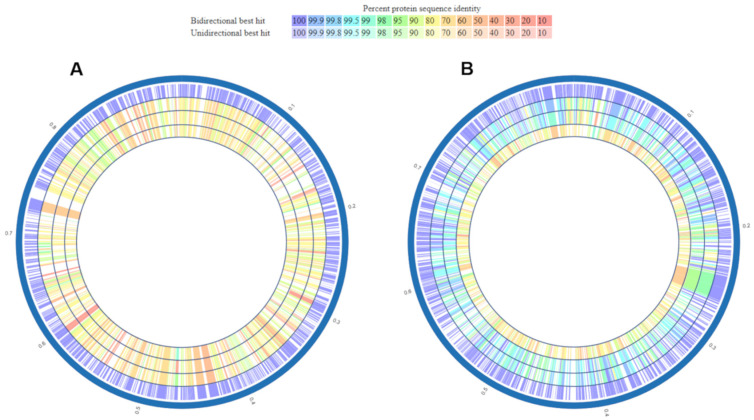
Proteome comparison utilizing the Proteome Comparison tool at PATRIC displaying proteins homologous to a protein of the reference genome *Mycoplasma* sp. 1654_15 (**A**) or *M. hyorhinis* JF5820 (**B**). The genomes are from outside to inside *Mycoplasma* sp. 1654_15, *M. hyorhinis* SK76, HUB-1, and JF5820 (**A**) or *M. hyorhinis* JF5820, SK76, HUB-1, and *Mycoplasma* sp. 1654_15 (**B**). The colour key indicates the percentage of protein sequence identity.

To analyse strain 1654_15 at the protein level, a proteome-wide comparison using average amino acid identity (AAI) profiler and Proteome Comparison tool at PATRIC was performed. AAI values between strain 1654_15 and *M. hyorhinis* SK76, HUB-1 and JF5820 were 81.79, 81.90, and 81.73%, respectively, not only demonstrating substantial differences between *Mycoplasma* sp. 1654_15 and *M. hyorhinis* at the protein level, but also supporting the previous finding of a limited resolution of the 16S rRNA gene between species (see above) if AAI values higher than 80% are obtained [32]. Differences at the protein level were further visualized by in silico proteome comparison tool at PATRIC demonstrating a similar degree (in %) of protein identity (largely 80%, yellow colour) between strain 1654_15 and *M. hyorhinis* SK76, HUB-1 and JF5820 (Figure 7).

**Figure 8:**
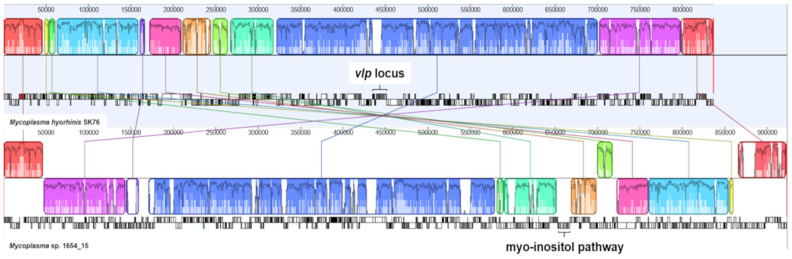
Genome alignment of strain 1654_15 and *M*. *hyorhinis* SK76 using progressive Mauve analysis. Co-linearity is represented by blocks of matching colour. Localization of the *vlp* locus and the myo-inositol pathway genes is indicated.

Genomic co-linearity of strain 1654_15 and *M. hyorhinis* SK76, HUB-1, and JF5820 was assessed by constructing chromosome alignments using progressive Mauve. Surprisingly, substantial co-linearity of strain 1654_15 and *M. hyorhinis* strains, with rearrangements or inversions occurring in large chromosomal blocks, were evident (shown for strain 1654_15 and *M. hyorhinis* SK76 in Figure 8) indicating that both species may have derived from a more recent common ancestor. However, genomic rearrangements with some losses (e.g., *vlp* locus) and several gains of genes (e.g., myo-inositol pathway) dispersed throughout the genome seem to have occurred during the evolution of *Mycoplasma* sp. 1654_15, leading to a specific repertoire of CDSs in its representative strain 1654_15.

**Figure 9:**
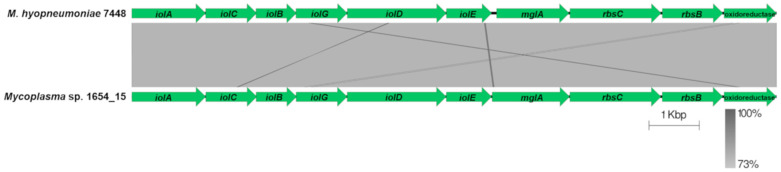
Organization of the myo-inositol pathway operon in strain 1654_15 and *M. hyopneumoniae* 7448. *iolA* = malonate-semialdehyde dehydrogenase gene, *ioC* = 5-keto-2-deoxygluconokinase gene, *iolB* = 5-deoxy-glucuronate isomerase gene, *iolG* = myo-inositol 2-dehydrogenase gene, *iolD* = 3D-(3,5/4)-trihydroxycyclohexane-1,2-dione hydrolase gene, *iolE* = inosose dehydratase gene, *mglA* = gene encoding the inositol transport system ATP-binding protein, *rbsC* = inositol transport system permease gene, *rbsB* = gene coding for the inositol transport system substrate-binding protein, oxidoreductase = gene encoding a non-specified Gfo/Idh/MocA family oxidoreductase.

**Figure 10:**
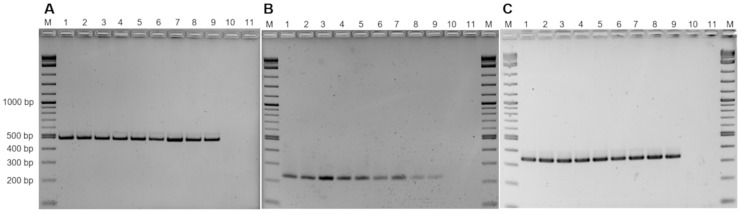
Presence of genes of the myo-inositol pathway in selected *Mycoplasma* sp. 1654_15 strains exemplified by the molecular detection of *iolE* (**A**), *iolA* (**B**), and *iolG* (**C**). Lane 1: strain 1654_6, 2: 1654_13, 3: 1654_15, 4: 2184_1, 5: 2184_3, 6: 2184_6, 7: 338_4, 8: 338_5; 9: 338_8, 10: negative control (water), 11: *M*. *hyorhinis* BTS-7^T^, M: molecular weight marker.

By employing the Protein Family Sorter tool at PATRIC unique CDSs solely present in 1654_15 or *M. hyorhinis* strains were identified. CDSs of *M. hyorhinis* genomes not shared with 1654_15 were, beside others, those of the variable lipoprotein (Vlp) system. In all three *M. hyorhinis* genomes the *vlp* locus was composed of seven distinct *vlp* genes (*vlpA*-*G*) whereas strain 1654_15 completely lacked a *vlp* locus. Most of the unique CDSs in the 1654_15 genome not shared with *M. hyorhinis* were annotated as hypothetical proteins, however, a genomic region comprising genes of the myo-inositol pathway (*iolA*, *iolB*, *iolC*, *iolD*, *iolE*, *iolG*, non-specific oxidoreductase–locus tag in CP051214: HF996_02950, _02940, _02945, _02930, _02925, _02905, and _02935; ABC inositol transporter genes *mglA*, *rpsB*, *rpsC*–HF996_02920, _02915, _02910) was shown to be encoded in strain 1654_15 only. Since myo-inositol metabolism was reported to be a species-specific trait potentially related to virulence of *M. hyopneumoniae* [33], the transcriptional units of myo-inositol catabolism in strain 1654_15 and *M. hyopneumoniae* 7448 were aligned using Easyfig presenting an identical organization structure of their myo-inositol catabolism operon, but with a mean reduced sequence identity of approximately 74% (Figure 9). All six key genes of the myo-inositol pathway (*iolA*, *iolB*, *iolC*, *iolD*, *iolE*, *iolG*) were also present in further isolates tested indicating that this pathway is a common metabolic feature of *Mycoplasma* sp. 1654_15 (Figure 10).

Further CDSs found in the genome of 1654_15 potentially related to virulence and pathogenicity of *Mycoplasma* sp. 1654_15 were, for example, a glycerol-3-phosphate oxidase (*glpO*, locus tag HF996_02520) shown to be involved in the production of cytotoxic H_2_O_2_ in other mycoplasmas [33,34], a predicted sialic acid scavenging and degradation pathway comprising two exo-alpha-sialidases (HF996_00850 and _00865), a N-acetylmannosamine-6-phsophate 2-isomerase (*nanE*, HF996_00590), a sialic acid transporter (HF996_00595), a N-acetylneuraminate lyase (*nanA*, HF996_00605), and a N-acetylmannosamine kinase (*nanK*, locus tag HF996_00610), a predicted immunoglobuline A1 proteases (HF996–00875), a glycosyl transferase (HF996_02470) possibly contributing to capsule formation [35], as well as two tandem pairs of *Mycoplasma* Ig binding (MIB, HF996_03795 and _03750)/*Mycoplasma* Ig protease (MIP, HF996_03790 and _03745) proteins in contrast to one pair found in *M. hyorhinis* [36].

## 3. Discussion

In common textbooks for swine diseases, eye diseases are described as multifactorial caused by a combination of infectious and abiotic factors. In pigs, conjunctivitis often results from systemic viral infections accompanied with other major symptoms [17]. Most frequently, PRRSV has been associated with conjunctivitis, however, it is unknown whether conjunctivitis is caused by specific viral pathomechanisms on mucosal surfaces or by secondary invaders inducing inflammation after viral pre-damage. PRRSV is supposed to cause local impairments at different organ sites but might not be the cause of a general immunosuppression [37]. An aggravation of lung alterations by dual infection with PRRSV and *M. hyorhinis* has been postulated [38]. The three farms examined in this study were positive for PRRSV, as are most of the farms in Germany [39,40]. In consequence, the involvement of PRRSV in causing the clinical picture observed cannot be excluded. Other common pathogens associated with conjunctivitis in pigs are *G. parasuis*, chlamydia, porcine cytomegalovirus, and swine influenza virus [17]. *G. parasuis* was detected during necropsy in some of the examined pigs but was not present in the conjunctival samples examined. Chlamydia was detected in some samples, and none of the pigs examined for swine influenza virus were positive, but examination for viral pathogens was not consistently performed on eye swabs, so that the presence of viruses cannot be excluded. Although multiple factors may have contributed to the observed eye disease several aspects suggest that *Mycoplasma* sp. 1654_15 has played a major role in disease establishment and progression including abundant isolation from the conjunctiva of affected pigs, close relationship to *M. hyorhinis* known to cause swine conjunctivitis, and identification of a panel of CDSs in the genome of strain 1654_15 potentially associated with virulence and pathogenicity.

A higher relevance for disease pathogenesis might be allocated to ammonia and dust than to other pathogens besides mycoplasmas. Detrimental effects of ammonia were shown in cell cultures [41,42], but also in several behavioral, experimental, and field studies [43,44,45,46]. Ammonia levels were determined to be beyond the 20-ppm legal threshold in all farms during the farm visit. Since several mycoplasmas grow best at pH 7.4–8.0 it may be speculated that ammonia can trigger the disease pathogenesis caused by mycoplasmas. However, if the tear fluid of pigs kept in buildings with increased ammonia concentrations is alkaline and thus optimized for mycoplasma growth needs to be investigated. Nevertheless, ammonia in air might produce a selection pressure for more specialized bacteria, resulting in bacterial evolution within the ecosystem of animal husbandry.

In farms A and B respiratory disorders were reported by the farmer, but the quantitative assessment of coughing and sneezing revealed no association with the observed eye disease. It can be assumed, that pigs suffered from unspecific inflammation in the upper (more sneezing) and lower (more coughing) respiratory tract and ammonia and dust might have been important trigger factors also for the development of respiratory symptoms on these farms. While a mean coughing index of ≥2.5% (threshold indicative for enzootic pneumonia [47]) was the exception and the sneezing index was always beyond 15% (threshold for healthy pigs kept in dry air conditions and common dust concentrations [48]), the published threshold of 10% for conjunctivitis in healthy pigs [49] was exceeded in farms B and C. In summary, the prevalence of conjunctivitis was not associated with the other clinical symptoms observed and the eye disease was thus considered as a separated disease picture.

A similar clinical presentation of swine conjunctivitis was recently reported by Resende et al. (2019) with clinical signs first observed in nursery pigs at the age of 8–10 weeks [16]. All age groups until week 22 were affected with similar high prevalence rates of 30–60%, and *M. hyorhinis* was detected by PCR in affected and unaffected pigs. Histological examination revealed the presence of *M. hyorhinis* in ulcerated conjunctival tissue using in situ hybridization, while cultivation of mycoplasmas failed. In our study the cultural isolation and quantification of mycoplasmas was successful from most conjunctival swabs, but localization of mycoplasmas in association with histological lesions was not performed due to the lack of an appropriate staining tool for species specific detection and identification of the novel mycoplasma species. Histological lesions present in one pig from farm A (Figure 1c) were mainly characterized as a lymphohistiocytic conjunctivitis and similar to those observed in previous studies [16,23].

Only two field cases of an eye disease in pigs associated with mycoplasma-like organisms and *M. hyorhinis*, respectively, have been published so far [16,23]. Furthermore, Friis (1976) reported the isolation of *M. hyorhinis*, *M. flocculare*, and an unidentifiable mycoplasma species serologically related to *M. hyorhinis* from the conjunctivae of piglets, providing first evidence that an undescribed mycoplasma species may exist and colonise the eyes of pigs [24]. Altogether, it cannot be excluded, that in all previous observations the new mycoplasma species described in our study has already been involved, including the most recent study in which *M. hyorhinis* was identified using real-time PCR and in situ hybridization targeting the 16S rRNA gene [16]. As previously shown for other closely related *Mycoplasma* species [28,30], our results of phylogenetic analysis strongly suggest that the 16S rRNA gene is insufficient to identify *Mycoplasma* sp. 1654_15 and, in consequence, diagnostics tests targeting the 16S rRNA gene may not accurately distinguish between the novel mycoplasma species and *M. hyorhinis*. In contrast, PCRs targeting unique gene sequences of the high affinity transport system protein p37 allowed the specific detection of *Mycoplasma* sp. 1654_15 and *M. hyorhinis* [50,51,52] in clinical specimens. Accurate identification of *Mycoplasma* sp. 1654_15 isolates was also achieved by MALDI-TOF MS providing that reference spectra of the novel mycoplasma species were included in the database. No ambiguous results or misidentification of *Mycoplasma* sp. 1654_15 isolates as *M. hyorhinis* were observed using MALDI-TOF MS confirming its excellence in the identification and differentiation of even closely related *Mycoplasma* species and its utility as screening method for novel mycoplasmas [25].

Both, partial *rpoB* sequencing and genome sequence-derived parameters applied for species delineation (ANI, AAI, TETRA, dDDH) clearly demonstrated that *Mycoplasma* sp. 1654_15 and *M. hyorhinis* represent phylogenetically closely related but distinct species within the Hyopneumoniae–Neurolyticum cluster of genus *Mycoplasma* [28,29,30,31,32]. Most striking differences between these two species were the lack of a *vlp* locus and the presence of myo-inositol pathway genes in *Mycoplasma* sp. 1654_15. Genes of the *vlp* locus encode phase and size variable surface lipoproteins (Vlp’s) [53] that play roles in adherence [54] and immune evasion [55], which appeared to be species-specific for *M. hyorhinis*. Conversely, genes coding for an entire myo-inositol pathway were present in *Mycoplasma* sp. 1654_15 isolates but missing in all *M. hyorhinis* genomes investigated. To date, catabolism of myo-inositol has only been reported in *M. hyopneumoniae* among mycoplasmas, implicated to be related to virulence and persistence of the pathogen in the porcine lung [33]. Since myo-inositol is freely available in the bloodstream of pigs, one could speculate that the highly vascularized conjunctivae with a microvasculature known to dilate in response to infection may provide *Mycoplasma* sp. 1654_15 with this alternative energy source, which could be essential for outcompeting other bacteria and for robust colonization of the conjunctival niche. However, whether the myo-inositol pathway is functional in *Mycoplasma* sp. 1654_15 remains to be elucidated and further experiments are required to support this hypothesis.

## 4. Materials and Methods

### 4.1. Clinical Examination on Farms and Sampling

One-time herd visits started in units with pigs of the youngest age group. Ammonia measurements were performed randomly in different production units, on the respective farm, using a transportable single-gas measuring device (Pac 8000 XXS NH_3_, Dräger, Lübeck, Germany). While in farm A the number of affected animals was not recorded, in farm B the proportion of animals in the mainly affected age group (9–10 weeks of life) was recorded, and in farm C proportions of animals with clinical symptoms in different age groups were quantified. Within three-minutes-time periods several swine groups in pens were examined in parallel by different observers to increase sample size during the time of the herd visit. Within the three-minutes-time periods the total number of animals in the inspected group was counted as well as the number of animals with conjunctivitis, the number of coughs and the number of sneezes. Coughing and sneezing indices were determined according to published methods with slight modification [47]. Pigs inside the pen to be examined were forced to get up to provoke coughing. Onset of coughing and sneezing was counted. During the three-minutes-time period single individuals showing coughing or sneezing were not differentiated. Two coughing bouts were counted, when coughing was absent between two coughing bouts for at least 10 s. The coughing and sneezing indices were calculated from total numbers of coughing and sneezing bouts divided by number of examined pigs × 3 (minutes observation time). For each group mean and median values were calculated.

Eye swabs were collected from pigs showing conjunctivitis. Single pigs were selected for necropsy for assessment of inner organ alterations. Pre-treatment with antimicrobial substances was unknown for animals necropsied. Eye alterations were characterized by histological examination of the eye globe and conjunctiva in one affected pig originating from farm 1.

### 4.2. Ethical Statement

Sampling of conjunctival swabs and necropsy of selected animals were routine diagnostic measures initiated by the swine practitioner responsible for herd health management in respective farms. Animal husbandry and diagnostic measures taken by the veterinarians were in accordance with the German Animal Welfare Law. Sampling and treatments were in accordance with standards of Good Clinical Practice. Since all samples were taken for routine diagnostic purposes with the consent of the owner, no ethical approval was required.

### 4.3. Characterization of Farms

Farm A: In this farrowing farm with 1200 sows, batch-wise farrowing took place in groups of 120 sows in 14 days intervals. During the suckling period of 21 days, piglets were vaccinated against *M. hyopneumoniae* in the first and third week of life, against the Porcine Reproductive and Respiratory Syndrome Virus (PRRSV) at day 14 of life, and against the Porcine Circovirus 2 (PCV2) in the third week of life. The only treatment was performed at day 5 of age, with a combined drug containing dihydrostreptomycinsulfat and benzylpenicillin-benzathin. The main concern of the farmer was respiratory symptoms like coughing and sneezing during nursery. Swellings around the eyes were recorded in many nursery pigs. During the herd visit, the anamnestically reported symptoms were confirmed. Measurements of ammonia concentrations in the air resulted in concentrations between 30–50 ppm NH_3_, which was beyond the legal limit in Germany of 20 ppm.

On farm 15, eye swabs and two nasal swabs from pigs showing conjunctivitis and nasal discharge were sampled for bacteriological examination. Four pigs with respiratory symptoms were sent for necropsy. Bacteriological examination was performed from different sites in the respiratory tract. Examination for *M. hyorhinis*, *M. hyopneumoniae*, PRRSV and influenza was performed by PCR.

Farm B: In this farrowing farm with 5000 sows, batch-wise farrowing took place in groups of 230–250 sows in weekly intervals. During the suckling period of 28 days, piglets were vaccinated against *M. hyopneumoniae* in the first and third week of life, against PCV2 in the third week of life, and against PRRSV at weaning. One day before weaning piglets were treated parenterally with tulathromycin against respiratory disorders and orally with colistin for 10 days against diarrhea. Starting from day 8, after weaning pigs were treated for 6 days orally with doxycycline against respiratory disorders, and with amoxicillin against *S. suis*-associated disease. The main concern of the farmer was coughing and conjunctivitis starting in the sixth week of life and aggravating until the end of nursery. During the herd visit the anamnestically reported symptoms were confirmed. Eight pens, each with 35 pigs at the age of 9–10 weeks, were examined during the farm visit. Measurements of ammonia concentrations in the air resulted in concentrations between 20–30 ppm NH_3_, which was beyond the legal limit in Germany of 20 ppm.

On farm B, eight eye swabs from pigs showing conjunctivitis were sampled. Four samples were taken from pigs at the age of 6 weeks, and four samples from pigs at the age of 10 weeks. Six pigs with respiratory symptoms were sent for necropsy. Bacteriological examination was performed from different sites in the respiratory tract. Examination for *M. hyopneumoniae*, PRRSV and influenza was performed by PCR. Lung tissue was used for cultivation of *M. hyorhinis*.

Farm C: This fattening farm with 3000 and 1500 fattening pigs at two locations got pigs at the age of 12 weeks from one farrowing farm with 500 sows located three kilometers apart. For several months, pigs had developed conjunctivitis within the first weeks after arrival. Mortality rate was 2.5%. In case of severe, purulent conjunctivitis pigs were treated parenterally with tulathromycin. Clinical examination was performed in the fattening farm and 12 pigs at the age of 12–20 weeks displaying conjunctivitis were sampled. To clarify, whether the eye disease already started during the nursery phase, also pigs in the farrowing farm were examined and five eye swabs were taken from pigs at the age of 5–8 weeks. Clinical examinations were performed in 30 pens in the nursery, and in 69 pens in the fattening farm. Measurements of ammonia concentrations in the air resulted in concentrations between 20–40 ppm NH_3_, which was beyond the legal limit in Germany of 20 ppm. Two pigs at the age of 12 weeks were sent for necropsy, only for assessment of macroscopic organ alterations and for PCV2 diagnostics.

### 4.4. Cultivation and MALDI-TOF Mass Spectrometry

Swab samples transported in Amies medium (Transwab^®^ Amies, Medical Wire and Equipment, Corsham, UK) were streaked onto Columbia agar with 5% sheep blood (BBL™, BD Diagnostics, Heidelberg, Germany) and Chocolate II agar plates (BBL™, BD Diagnostics, Heidelberg, Germany) incubated at 37 °C in 5% CO_2_ atmosphere for 48 h. Bacterial colonies isolated were identified by MALDI-TOF MS (Bruker Daltonik GmbH, Bremen, Germany), as described previously [56]. For the isolation of mycoplasmas, swabs were placed into one mL 2-SP medium containing 48.5 g/L sucrose in phosphate buffer (1.1 g/L mono- and 2.1 g/L dibasic potassium phosphate, pH = 7.0) supplemented with 10% fetal calf serum, vortexed, and diluted in 2-SP medium up to 1 × 10^−5^. One hundred µL of each dilution was transferred onto modified SP4 agar, as well as into a modified SP4 broth medium [57], to increase the probability of isolation, both incubated at 37 °C in 5% CO2 atmosphere for up to 10 days. When a colour change of the broth medium was observed, 100 µL of cultures were immediately transferred onto modified SP4 agar. Agar plates were daily checked for mycoplasma colony formation using a stereo microscope. For the identification of mycoplasmas isolated, single colonies were picked and re-cultured in a modified SP4 medium to establish pure colony-derived cultures. Mycoplasma cells were then harvested from 1 mL of late log-phase cultures and identified by MALDI-TOF MS, as previously described [25] using a microflex LT Biotyper operating system (Bruker Daltonik GmbH, Bremen, Germany) and a large in-house library containing 810 mycoplasma reference spectra, including all cultivable porcine mycoplasmas. Since mycoplasmas isolated from farm A remained undiagnosed by MALDI-TOF MS, MSPs of one selected isolate from each farm (farm A–strain 1654_15, farm B–strain 2184_1, and farm C–strain 338_4) were generated and included in the in-house mycoplasma reference spectrum library [25].

### 4.5. Determination of MIC Values

MICs of the following antimicrobial agents, against three selected mycoplasma isolates from each farm (Table 1), were determined as previously described [26,58]: tetracycline–oxytetracycline (tested range of twofold diluted concentrations: 0.25–16 µg/mL), aminoglycoside–gentamicin (0.25–16 µg/mL), aminocyclitol–spectinomycin (0.5–64 µg/mL), macrolides–tylosin tartrate, tilmicosin, and tulathromycin (all 0.5-64 µg/mL), lincosamide–lincomycin (0.25–32 µg/mL), phenicol–florfenicol (0.25–16 µg/mL), fluoroquinolone–enrofloxacin (all 0.25–16 µg/mL), and pleuromutilin–tiamulin (0.25–16 µg/mL). *M. hyorhinis* BTS-7^T^ was included in the study as quality control strain.

### 4.6. PCR Analysis of Swab Samples and Mycoplasma Cultures

Swab samples without transport medium (Dryswab™, Medical Wire and Equipment, Corsham, UK) as well as one ml of pure mycoplasma cultures were subjected to DNA extraction using GenElute™ Mammalian Genomic DNA Miniprep Kit (Sigma–Aldrich, Vienna, Austria) according to the manufacturer’s instructions. For detection of chlamydia in swab samples, a PCR targeting the *omp2* gene followed by amplicon sequencing was employed [59]. *M. hyorhinis* was detected by PCR targeting a species-specific fragment of the *p37* gene [50] which did not amplify the novel mycoplasma species. Based on major sequence differences in the upstream region of the *p37* genes of *M. hyorhinis* and the fully sequenced strain 1654_15 isolated in this study, a specific PCR was established for the detection of the novel mycoplasma species in swab samples and to confirm species identification of isolates obtained by MALDI-TOF MS (Table 1 and Table 3). Furthermore, PCRs targeting *iolA*, *iolB*, *iolC*, *iolD*, *iolE*, and *iolG* were established to confirm the presence of six key genes of the myo-inositol pathway in mycoplasma isolates that were selected for further analyses (Table 3). Target-specific primers were designed from the complete genome sequence of strain 1654_15 using Geneious Prime 2020.1.2 (Biomatters Ltd., Auckland, New Zealand). For all newly established PCRs, the reaction mixtures consisted of 30 µL OneTaq^®^ Quick-Load^®^ 2X Master Mix with Standard Puffer (New England Biolabs Inc., Ipswich, USA), 1 µL (32 pmol) of each primer (Table 3), 2.5 µL of DNA, and 23 µL PCR grade water. For amplification, the following protocol was employed: initial denaturation at 94 °C for 2 min, followed by 35 cycles of denaturation at 94 °C for 1 min, annealing at 55 °C for 30 sec, and elongation at 72 °C for 1 min, and final extension at 72 °C for 5 min. DNA from strain 1654_15 was used as positive control, negative control samples included DNA from *M. hyorhinis* BTS-7^T^ or PCR-grade water. Amplicons were sequenced at LGC Genomics, Berlin, Germany.

### 4.7. Phylogenetic Analysis of Selected Mycoplasma Isolates

16S rRNA and partial *rpoB* genes of three mycoplasma isolates randomly selected from each case were amplified as previously described using primer sets 27f/1492r [60] and rpoB-F-MYC/rpoB-R-MYC [28], respectively. Amplicons were purified employing GenElute Purification kit (Sigma–Aldrich, Vienna, Austria) and Sanger sequenced in both directions at LGC Genomics, Berlin, Germany. Similarities of 16S rRNA gene sequences were determined using EzBioCloud [61]. For partial *rpoB* sequences a BLAST search to GenBank entries was performed [62]. Sequences were aligned using Clustal W [63] and phylogenetic trees comprising all members of the Hyopneumoniae–Neurolyticum phylogenetic cluster of genus *Mycoplasma* were constructed in MEGA 6.0 using the maximum-likelihood algorithm with Kimura 2-parameter adjustment and 1000 bootstrap replications [64].

### 4.8. Genome Sequencing of Strain 1654_15

A single colony of strain 1654_15 was picked and grown to the mid-log phase in 50 mL modified SP4 medium and pelleted by centrifugation at 20,000× *g* for 10 min. Genomic DNA was then isolated from the pellet using DNeasy blood and tissue kit (Qiagen GmbH, Hilden, Germany) according to the manufacturer’s instruction. Quantity and quality of extracted genomic DNA were determined by NanoDrop™ 2000 (Fisher Scientific GmbH, Schwerte, Germany). For MinION sequencing (Oxford Nanopore Technologies, Oxford, UK) the Rapid Sequencing Kit SQK-RAD004 was applied for library preparation and 1D sequencing was performed for 48 h using a FLO-MIN106D R9.4.1 flow cell. Real-time base calling was performed using MinKNOW operating software (version 3.6.0) with integrated Guppy data processing tool (version 3.2.8) for adapter trimming (Oxford Nanopore Technologies). Produced FASTQ files were processed with Geneious Prime 2020.1.2 (Biomatters Ltd.) to remove sequences shorter than 3000 bases. Genomic DNA of *Mycoplasma* sp. 1654_15 was additionally sequenced on an Illumina MiSeq sequencer. Paired reads were created from raw data using Geneious Prime 2020.1.2 (Biomatters Ltd.) and trimmed by BBDuk plugin (version 38.37) with filtering for Phred quality values above 30, and a minimal read length of 100 bases after removal of the adapter sequences. Genome assembly of Illumina short reads along with MinION long reads was performed in hybrid assembly mode of the Unicycler pipeline version 0.4.8 (https://github.com/rrwick/Unicycler/) with default settings [65]. Finally, the completed and circularized genome of *Mycoplasma* sp. 1654_15 was automatically annotated using the National Center for Biotechnology Information (NCBI) Prokaryotic Genome Annotation Pipeline (PGAP) and the Rapid Annotations using Subsystems Technology (RAST) (https://rast.nmpdr.org/).

### 4.9. Genome Comparison Analysis

Reference genomes of *M. hyorhinis* SK76, HUB-1, and JF5820 as well as *M. hyopneumoniae* 7448 used for comparative studies were compiled from the NCBI GenBank database (accession numbers: SK76–CP003914, HUB-1–CP002170, JF5820–CP035041, and 7448–AE017244) or from PATRIC [66]. Average nucleotide identity based on BLAST algorithm (ANIb) or MUMmer (ANIm) and tetranucleotide signature correlation index (TETRA) scores were calculated using the JSpeciesWS online tool [67]. In silico digital DNA–DNA hybridization (dDDH) values were determined using the Genome-to-Genome Distance Calculator (GGDC version 2.1; http://ggdc.dsmz.de) by applying recommended formula 2 [68]. Average amino acid identity (AAI) scores were assessed using AAI calculator (http://enve-omics.ce.gatech.edu/aai/index) [32]. In addition, a phylogenetic tree was constructed from the genomes of strain 1654_15 and selected members of the Hyopneumoniae–Neurolyticum cluster using the Codon Tree method at PATRIC, RAxML version 8.2.11, and Fast Bootstrapping [69,70,71]. Two methodologies available in PATRIC were further used to compare genomes including Protein Family Sorter [72] to identify unique CDSs or regions in individual genomes. Unique CDSs or regions identified were then verified by BLAST against representative databases at NCBI. Annotation in PATRIC assigned protein families that are scoped at the genus level (PLfams, PGfams) were compared in a bi-directional, best BLASTP hit analysis using Proteome Comparison tool, and visualized by the circus tool at PATRIC [72,73]. To provide consistency across results, only genomes annotated with Rapid Annotation using Subsystem Technology tool kit RASTtk [74] at PATRIC were used for these analyses. In addition, genomes were aligned using progressive Mauve analysis [75] available as a subprogram in Geneious Prime 2020.1.2 (Biomatters Ltd.). Furthermore, the transcriptional units of myo-inositol catabolism (annotated with Prokaryotic Genome Annotation Pipeline (PGAP) [76]) in strain 1654_15 and *M. hyopneumoniae* 7448 were aligned using Easyfig [77].

### 4.10. Accession Number

The complete genome sequence of *Mycoplasma* sp. 1654_15 is available at NCBI under the accession number CP051214.

## 5. Conclusions

In conclusion, a novel mycoplasma species, tentatively named *Mycoplasma* sp. 1654_15 and associated with swine conjunctivitis in three unrelated German farms has been identified and characterized. Although the genome of strain 1654_15 has been shown to share a considerable number of genes with *M. hyorhinis*, phylogenetic analyses, and genome sequence-derived parameters clearly demonstrated that both species represent closely related but distinct taxa within genus *Mycoplasma*. Lack of the *vlp* locus and presence of an entire myo-inositol pathway in *Mycoplasma* sp. 1654_15 were the two most prominent traits differentiating the novel mycoplasma species from *M. hyorhinis*. Based on the abundant isolation of the new mycoplasma species from conjunctival swabs of affected pigs, its close relationship to *M. hyorhinis*, and the identification of a panel of CDSs potentially associated with virulence and pathogenicity we suggest the involvement of *Mycoplasma* sp. 1654_15 in the pathogenesis of the reported eye disease. However, the pathogenicity of *Mycoplasma* sp. 1654_15 has to be investigated and confirmed by fulfilling Koch’s postulates in further studies to support our conclusion that the novel mycoplasma species is a primary pathogen causing swine conjunctivitis.

## Figures and Tables

**Table 1:** Conjunctival samples investigated, and results obtained by cultivation and PCR.

Sample ID	Farm	Geography	Collection Date (Month/Year)	Mycoplasmas (Culture)	*Mycoplasma* sp. 1654_15-Specific PCR (*p37*)	*M. hyorhinis*-Specific PCR (*p37*)	*Chlamydia* spp.-Specific PCR	Bacteria (Culture)
1654_1	A	Lower Saxony	4/2018	***Mycoplasma* sp.** **(ID 1654_1)**	**positive**	negative	negative	negative
1654_2	A	Lower Saxony	4/2018	**Positive ^b^**	**positive**	negative	negative	*Escherichia coli*
1654_3	A	Lower Saxony	4/2018	**Positive ^b^**	**positive**	negative	negative	*St. chromogenes*
1654_4	A	Lower Saxony	4/2018	***Mycoplasma* sp.** **(ID 1654_4)**	**positive**	negative	**positive**	negative
1654_5	A	Lower Saxony	4/2018	**Positive ^b^**	**positive**	negative	negative	*E. coli*
1654_6	A	Lower Saxony	4/2018	***Mycoplasma* sp.** **(ID 1654_6) ^a^**	**positive**	negative	negative	negative
1654_7	A	Lower Saxony	4/2018	***Mycoplasma* sp.** **(ID 1654_7)**	**positive**	**positive**	negative	negative
1654_8	A	Lower Saxony	4/2018	***Mycoplasma* sp.** **(ID 1654_8)**	**positive**	negative	**positive**	negative
1654_9	A	Lower Saxony	4/2018	***Mycoplasma* sp.** **(ID 1654_9)**	**positive**	negative	negative	negative
1654_10	A	Lower Saxony	4/2018	negative	**positive**	negative	negative	negative
1654_11	A	Lower Saxony	4/2018	**positive^b^**	**positive**	negative	negative	*St. chromogenes*
1654_12	A	Lower Saxony	4/2018	***Mycoplasma* sp.** **(ID 1654_12)**	**positive**	positive	**positive**	negative
1654_13	A	Lower Saxony	4/2018	***Mycoplasma* sp.** **(ID 1654_13) ^a^**	**positive**	negative	negative	negative
1654_14	A	Lower Saxony	4/2018	**Positive ^b^**	**positive**	negative	negative	*E. coli*
1654_15	A	Lower Saxony	4/2018	***Mycoplasma* sp.** (ID 1654_15) * ^a^	**positive**	negative	negative	negative
2184_1	B	North Rhine-Westphalia	11/2018	***Mycoplasma* sp.** **(ID 2184_1) ^a^**	**positive**	negative	negative	negative
2184_2	B	North Rhine-Westphalia	11/2018	***Mycoplasma* sp.** **(ID 2184_2)**	**positive**	negative	negative	negative
2184_3	B	North Rhine-Westphalia	11/2018	***Mycoplasma* sp.** **(ID 2184_3) ^a^**	**positive**	negative	negative	negative
2184_4	B	North Rhine-Westphalia	11/2018	***Mycoplasma* sp.** **(ID 2184_4)**	**positive**	negative	negative	negative
2184_5	B	North Rhine-Westphalia	11/2018	***Mycoplasma* sp.** **(ID 2184_5)**	**positive**	negative	**positive**	negative
2184_6	B	North Rhine-Westphalia	11/2018	***Mycoplasma* sp.** **(ID 2184_6) ^a^**	**positive**	**positive**	**positive**	negative
2184_7	B	North Rhine-Westphalia	11/2018	**Positive ^b^**	**positive**	negative	negative	*E. coli*
2184_8	B	North Rhine-Westphalia	11/2018	negative	**positive**	negative	negative	negative
338_1	C	North Rhine-Westphalia	1/2020	***Mycoplasma* sp.** **(ID 338_1)**	**positive**	**positive**	**positive**	negative
338_2	C	North Rhine-Westphalia	1/2020	***Mycoplasma* sp.** **(ID 338_2)**	**positive**	negative	negative	negative
338_3	C	North Rhine-Westphalia	1/2020	***Mycoplasma* sp.** **(ID 338_3)**	**positive**	negative	negative	negative
338_4	C	North Rhine-Westphalia	1/2020	***Mycoplasma* sp.** **(ID 338_4) ^a^**	**positive**	negative	**positive**	negative
338_5	C	North Rhine-Westphalia	1/2020	***Mycoplasma* sp.** **(ID 338_5) ^a^**	**positive**	negative	negative	negative
338_6	C	North Rhine-Westphalia	1/2020	***Mycoplasma* sp.** **(ID 338_6)**	**positive**	negative	negative	*St. hyicus*
338_7	C	North Rhine-Westphalia	1/2020	***Mycoplasma* sp.** **(ID 338_7)**	**positive**	negative	negative	*St. hyicus*
338_8	C	North Rhine-Westphalia	1/2020	***Mycoplasma* sp.** **(ID 338_8) ^a^**	**positive**	negative	negative	negative
338_9	C	North Rhine-Westphalia	1/2020	***Mycoplasma* sp.** **(ID 338_9)**	**positive**	negative	negative	negative
338_10	C	North Rhine-Westphalia	1/2020	***Mycoplasma* sp.** **(ID 338_10)**	**positive**	**positive**	negative	negative
338_11	C	North Rhine-Westphalia	1/2020	***Mycoplasma* sp.** **(ID 338_11)**	**positive**	negative	**positive**	negative
338_12	C	North Rhine-Westphalia	1/2020	***Mycoplasma* sp.** **(ID 338_12)**	**positive**	negative	negative	negative

* selected for whole genome sequencing, ^a^ selected for further analyses (determination of minimum inhibitory concentration (MIC)values, detection of genes of the myo-inositol pathway, phylogenetic analysis), ^b^ culturally positive for mycoplasmas but species identification was prevented by bacterial overgrowth, *E*. *Escherichia*, *St*. *Staphylococcus.*

**Table 2:** MIC values of nine selected *Mycoplasma* sp. 1654_15 isolates (3 from each farm) and *M. hyorhinis* BTS-7^T^ in µg/mL.

Mycoplasma Isolate/Strain	TYLT	TIL	TUL	TIA	OXY	GEN	SPE	LINC	FFN	ENR
1654_6	8	>64	>64	≤0.25	0.5	1	4	2	2	4
1654_13	8	>64	>64	≤0.25	0.5	1	4	2	2	4
1654_15	8	>64	>64	≤0.25	0.5	1	4	2	2	4
2184_1	16	>64	>64	≤0.25	0.5	2	4	4	2	8
2184_3	16	>64	>64	≤0.25	0.5	2	4	4	2	8
2184_6	16	>64	>64	≤0.25	0.5	2	4	4	2	8
338_4	16	>64	>64	≤0.25	0.5	1	4	2	2	4
338_5	16	>64	>64	≤0.25	0.5	1	4	2	2	4
338_8	16	>64	>64	≤0.25	0.5	1	4	2	2	4
BTS-7^T^	≤0.25	1	2	≤0.25	≤0.25	1	4	≤0.25	1	0.50

TYLT: tylosin tartrate, TIL: tilmicosin, TUL: tulathromycin, TIA: tiamulin, OXY: oxytetracycline, GEN: gentamicin, SPE: spectinomycin, LINC: lincomycin, FFN: florfenicol, ENR: enrofloxacin.

**Table 3:** Primers used for specific detection of the novel mycoplasma species (*p37*) and six genes of the myo-inositol pathway (*iolA*, *iolB*, *iolC*, *iolD*, *iolE*, *iolG*).

Primer	Sequence	Product Size
*p37*-F	5′-TTTCACCGGCAGACTGAGAC-3′	322 bp
*p37*-R	5′-GCTGGAGTCACAACATCTGGA-3′	
*iolA*-F	5′-GCTGCTGTTTCAATGGGAGC-3′	225 bp
*iolA*-R	5′-AGCAGGATTACCAAGCGGAA-3′	
*iolB*-F	5′-ACAAGTGCTCTTCTGCTTCGA-3′	239 bp
*iolB*-R	5′-ACATCACCATCCACAGCCTG-3′	
*iolC*-F	5′-CACCGCCACCGTATCCTTTT-3′	696 bp
*iolC*-R	5′-ACATCGGAGGATCAACTGCA-3′	
*iolD*-F	5′-AGCGCAGAACTAGCTTGTGA-3′	464 bp
*iolD*-R	5′-GCAGCGAATATGCTAACCGC-3′	
*iolE*-F	5′-ATGTCTTCACCTGTGTGGG-3′	478 bp
*iolE*-R	5′-CACAAGCGGGCTATCAAGGA-3′	
*iolG*-F	5′-AGTCCAACAGCACATCATCCA-3′	333 bp
*iolG*-R	5′-TAAATCGTGCGCTCCGACAT-3′	

## Data Availability

The data presented in this study are available in this article and Supplementary Material.

## Supplementary Materials

The following are available online at https://www.mdpi.com/2076-0817/10/1/13/s1, Figure S1: Identification of a mycoplasma isolate using MALDI-TOF MS.
