# Supplementary material for: Swine Conjunctivitis Associated with a Novel Mycoplasma Species Closely Related to Mycoplasma hyorhinis
Source: Pathogens. 2020 Dec 25;10(1):13. doi: 10.3390/pathogens10010013 (PMC7824142; doi:10.3390/pathogens10010013)
Supplement: Supplementary file 1 [file pathogens-10-00013-s001.zip › pathogens-987866supp.docx]

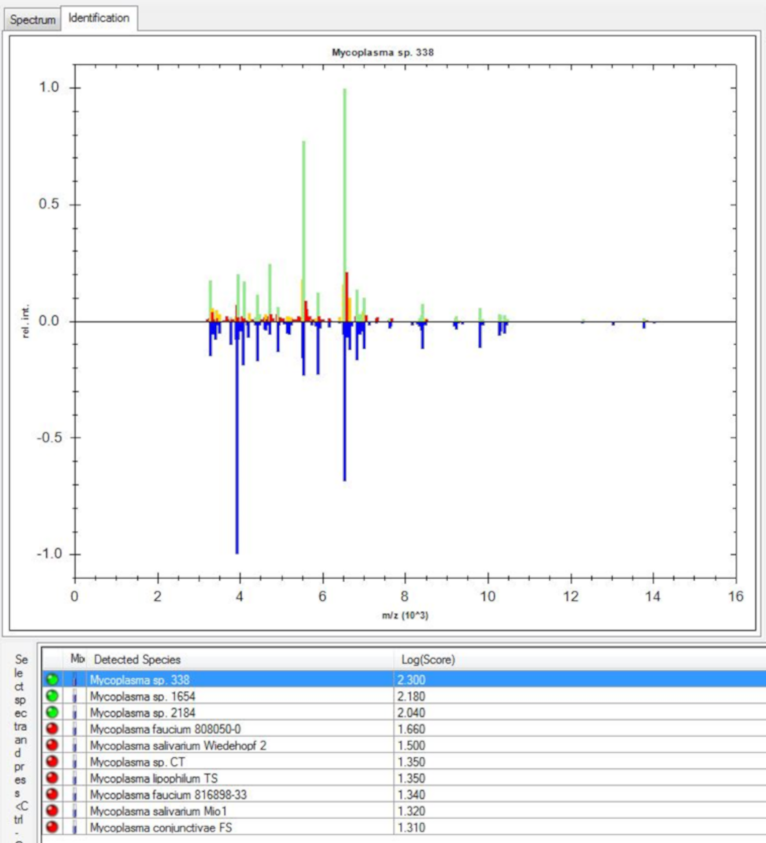


**Figure S1.** Identification of a mycoplasma isolate using MALDI-TOF MS producing log scores above 2.00 to three representatives of *Mycoplasma* sp. 1654_15.
